# Accelerated Discovery of Graphene Kirigami with an Enhanced Elastocaloric Effect via Machine Learning

**DOI:** 10.1021/acs.nanolett.5c05140

**Published:** 2026-01-19

**Authors:** Franklin F. da Silva Filho, Luiz Felipe C. Pereira

**Affiliations:** Departamento de Física, Centro de Ciências Exatas e da Natureza, 28116 Universidade Federal de Pernambuco, Recife 50670-901, Brazil

**Keywords:** graphene, kirigami, elastocaloric effect, machine learning

## Abstract

Recent studies have examined the elastocaloric response of graphene kirigami (GK) and shown how it may be tailored through geometric design. This tunability makes GK a promising platform for applications in nanoscale solid-state thermal devices. In this work, we combine molecular dynamics (MD) simulations and machine learning (ML) to explore how GK geometries affect the elastocaloric coefficient (ECC), defined as the adiabatic ratio between temperature change and applied tensile stress. A data set of 16,807 GK configurations was generated through systematic cut patterns and evaluated via MD at room temperature. Using this data, both classical and deep-learning models were trained, with a convolutional neural network (CNN) achieving the best performance (RMSE = 0.064 K GPa^–1^; *R* ^2^ = 0.96). Model-guided optimization identified high-ECC designs 10 times faster than random search, demonstrating the power of ML-assisted strategies for the accelerated discovery of advanced elastocaloric materials.

## Graphical abstract

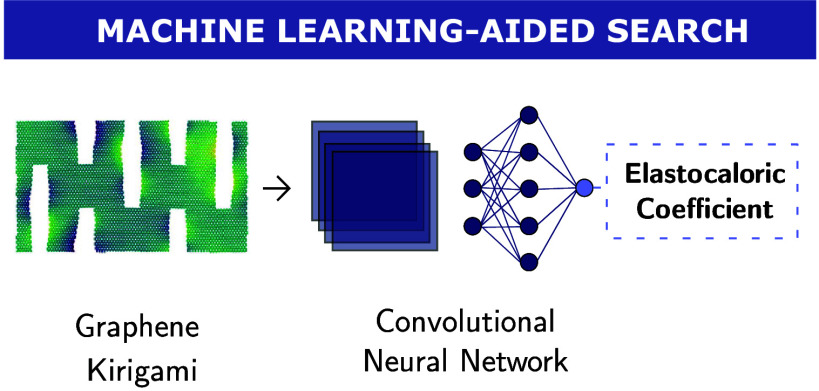

Two-dimensional (2D) materials, particularly graphene, have captivated scientific interest due to their extraordinary properties at the atomic scale. Since its experimental isolation in 2004,^1^ graphene has been extensively studied for its high tensile strength, excellent thermal and electrical conductivity.^2–4^ The versatility of graphene has led to a wide range of derivatives and geometrically engineered modifications, including graphene oxide,^5^ graphyne,^6^ graphdyne,^7^ graphene nanomeshes,^8^ and superlattices,^9–11^ all of which can be applied to tailor graphene’s intrinsic properties for specialized applications.

One promising structural paradigm is *graphene kirigami* (GK), where precise cuts are introduced into the graphene lattice to form complex architectures inspired by the Japanese art of paper cutting.^12^ Recent experimental advances have demonstrated that GK can be fabricated through top-down patterning strategies such as photolithography and plasma etching, enabling precise control over cut geometry. These fabrication methods have yielded GK specimens capable of sustaining extreme tensile deformations, with reported ultimate strains approaching ∼370% and exhibiting high fatigue tolerance under cyclic loading up to ∼70% strain.^12,13^ Moreover, specific kirigami motifs can function as nanoscale mechanical elements: depending on their geometry, GK structures can display effective elastic constants spanning orders of magnitude (from 1 to ∼10^–9^ N m^–1^). Furthermore, the geometric deformation mechanisms in GK allow large reversible strains while preserving key physical properties. Experiments have shown that certain patterns maintain nearly constant electrical conductance throughout the stretching process,^12^ a behavior difficult to achieve through conventional materials engineering strategies. These kirigami structures significantly alter the mechanical response of graphene, enabling high stretchability, strain localization, and tunable stiffness. Such geometric modifications hold potential in diverse fields, including flexible electronics, strain-engineered devices, stretchable conductors, and nanoactuators.^13–17^ This unique combination of tunable mechanics, structural robustness, and functional stability underscores the relevance of GK for nanoscale applications.

In addition to altering the mechanical flexibility of graphene, kirigami patterns may also allow control of thermomechanical properties, including elastocaloric effects (ECEs).^18^ The ECE is a mechanism in which a material undergoes a reversible temperature change upon a change in strain of the material due to a compressive or tensile stress, under adiabatic conditions. Traditionally studied in shape-memory alloys and ferroelectric materials,^19^ this effect has recently gained traction on the nanoscale, where unique size-dependent behaviors can emerge. In the context of energy-efficient cooling and thermal regulation, especially in micro- and nanoscale devices where traditional refrigeration methods are impractical, elastocaloric materials provide an appealing alternative. Several coefficients can be used to evaluate the elastocaloric performance, and among them the *field-normalized elastocaloric coefficient*

1 $$ECC=\frac{\Delta T}{\Delta \sigma }$$

defined as the adiabatic temperature change (Δ*T*) per unit of stress (Δσ), is a good candidate for evaluating materials with various structural or geometric characteristics. This normalization facilitates the design of architectures that maximize the thermal response per unit of applied load.^20^

To investigate the ECE in low-dimensional systems, molecular dynamics (MD) simulations can provide a powerful atomistic framework to resolve structural responses under mechanical loading.^21^ MD enables the direct evaluation of temperature variations, stress fields, and microscopic rearrangements within well-defined thermodynamic ensembles, making it a powerful tool for quantifying nanoscale phenomena. However, such simulations can be computationally demanding when applied to large design spaces or when repeated across several structural configurations. Machine learning (ML) offers a complementary strategy to overcome those challenges.^22,23^ By learning structure–property relationships from a representative set of MD simulations, ML models can serve as surrogate predictors, enabling rapid screening of candidate architectures at a fraction of the computational cost.^24^ In this integrated MD–ML framework, MD supplies physically grounded data while ML accelerates exploration of high-dimensional design spaces, enabling efficient identification of architectures with optimal properties.^25–28^

The elastocaloric response has been investigated in pristine graphene,^29,30^ carbon and boron nitride nanotubes,^31–33^ and graphynes^34^ through MD simulations. Regarding GK, thermal and electronic transport characteristics were shown to be tunable by periodic patterns of linear and curved cuts.^35^ More recently, the ECE was demonstrated in a specific GK design, including its application in an Otto-like thermodynamic cycle.^36^ However, a systematic exploration of how kirigami geometry influences the ECE is still necessary. Previous studies have generally examined isolated or manually designed structures, without addressing the vast combinatorial space of possible cut configurations. Because the number of potential designs grows exponentially with grid resolution, exhaustive simulation-based searches are computationally prohibitive. ML-assisted approaches have recently proven effective for identifying optimal stretchability in GK,^37^ underscoring the need for intelligent strategies that integrate physical simulations with predictive models to guide design optimization.

In this study, we investigate kirigami structures that were derived from a pristine graphene sheet measuring 200 × 117 Å, containing 8,640 carbon atoms. The sheet was oriented such that the Cartesian *x* axis corresponded to the zigzag direction, while the *y* axis was aligned with the armchair direction. The sheet was then partitioned into a 3 × 5 grid, with each cell measuring approximately 40 × 39 Å. Within each grid cell, a vertical cut defect could be introduced, aligned with the *y* direction and measuring 13.3 × 39 Å, or left pristine. An example of a representative GK can be seen in Figure 1.

**Figure 1:**
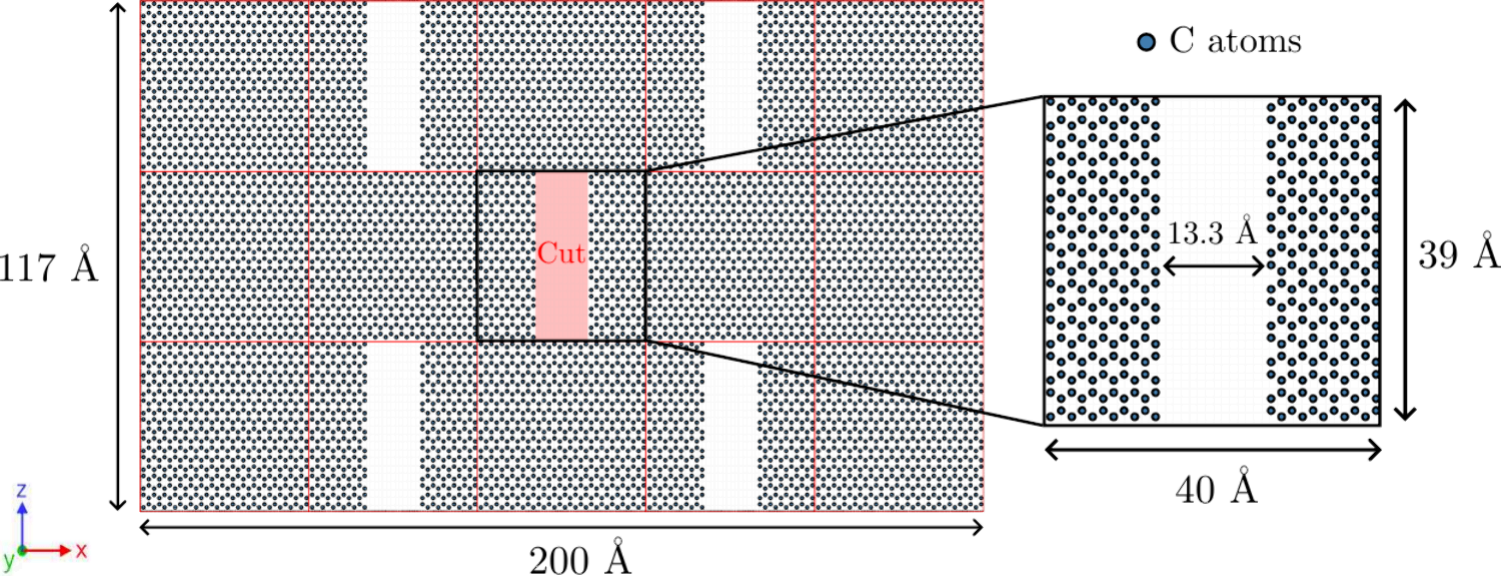
Representative GK. The *x* axis aligns with the zigzag direction, along which tensile loading is applied. Red grids indicate possible cut regions (red rectangle), and circles represent carbon atoms.

The binary choice for each of the 15 cells resulted in 2^15^ = 32, 768 possible configurations. However, configurations containing three vertically aligned cuts that completely severed the structure along the *y*-direction were excluded. This restriction reduced the total number of valid configurations to 16,807, with the number of cut defects per structure ranging from 0 (pristine graphene) to 10. Considering periodic boundary conditions (PBCs) applied in both *x* and *y* directions, structures that can be transformed into one another by a simple translation within the *xy* plane must be considered equivalent. We refer to these as belonging to the same *canonical class*. Therefore, considering this symmetry, the 16,807 valid structures were reduced to 1,123 canonical configurations. Only these canonical configurations require MD simulations, since the behavior of each equivalence class is fully captured by a single representative. The MD-derived mechanical properties of each canonical configuration were then propagated to all members of its equivalence class, ensuring that every one of the 16,807 valid designs is assigned consistent physical properties.

MD simulations were carried out using Large-scale Atomic Molecular Massively Parallel Simulator (LAMMPS)^38^ and the interatomic forces were described using the Tersoff bond-order potential,^39^ which has been extensively employed in previous studies to model mechanical and thermal properties of graphene and related nanostructures.^11,21,40^ We adopted the reparameterized form proposed by Lindsay and Broido^41^ and adjusted the potential cutoff parameter *R* from 1.95 to 2.15 Å to mitigate unphysical strain hardening as observed in previous studies.^11^ Prior to evaluating the kirigami structures, we validated the chosen interatomic potential by simulating pristine graphene under uniaxial tension. The resulting elastic modulus (947 ± 17 GPa), ultimate tensile strength (120 ± 10 GPa), and failure strain (0.22 ± 0.02) are in good agreement with established computational and experimental benchmarks.^21^ In particular, these values closely match the first experimental measurements reported using AFM nanoindentation, which obtained an elastic modulus of 1.0 ± 0.1 TPa, an ultimate tensile strength of 130 ± 10 GPa, and a failure strain of approximately 0.25.^42^ This consistency supports the suitability and reliability of the potential employed in the present study.

Additionally, Table S1 reports the ECC obtained for pristine graphene and a representative GK under both finite and PBCs, and for two system sizes. The comparison shows that the ECC values computed using periodic boundaries exhibit compatible results with increasing system dimensions, indicating that our chosen simulation cell is sufficiently large to yield size-converged estimates of the elastocaloric response. In contrast, simulations employing finite boundaries display quantitative deviations from their periodic counterparts, a result that is also consistent with previously reported studies.^34^ Therefore, the periodic setup enables reliable predictions while allowing the use of smaller simulation domains, which is essential for the extensive exploration of the GK configurational space carried out in this work.

The structures were first subjected to an energy minimization at zero in-plane stress using a conjugate gradient algorithm with a force tolerance of 10^–6^ eV Å^–1^. Thermal equilibration was then carried out in the isothermal–isobaric (*NPT*) ensemble at 300 K and zero in-plane stress for 500 ps, using a Nosé–Hoover thermostat and barostat with a time step *dt* = 0.1 fs. After equilibration, the ECC was obtained in a microcanonical (*NVE*) ensemble by applying a uniaxial deformation along the *x*-direction at a constant strain rate of 0.2% ps^–1^, up to a maximum strain of 10%, and using a time step of *dt* = 0.025 fs. We utilized an effective thickness of 3.35 Å to calculate the stress in the *x* direction (σ_*xx*_), and the strain ε was defined as

2 $$\varepsilon =\frac{l_{x}-l_{0}}{l_{0}}$$

where *l*_*x*_ and *l*_0_ are the current and equilibrated length in the *x* direction, respectively. The elastocaloric coefficient was calculated as ECC = Δ*T*/Δσ, using average temperature and stress values within the first and last 0.2% strain windows. Each simulation was repeated five times with a different random seed to set the initial atomic velocities, and obtained results are given as averages. Atomic coordinate manipulation and visualization were performed with the Atomic Simulation Environment (ASE) library^43^ and OVITO.^44^

We begin our discussion comparing the results of pristine graphene with a selected GK design. In Figure 2a, we show that pristine graphene exhibits a stress of approximately 80 GPa at about 10% strain, in agreement with previous reports on its high in-plane stiffness.^45^ In contrast, the GK structure shows a markedly different mechanical response, reaching only about 5 GPa at the same strain level, consistent with its cut-enabled compliance observed in similar designed materials.^40^ Figure 2b shows that pristine graphene displays a negative ECE, with a temperature drop of approximately Δ*T* = – 16 K, while GK exhibits a positive temperature variation of about Δ*T* = +9 K. These contrasting trends highlight the ability of kirigami patterns to fundamentally alter both the mechanical response and the elastocaloric behavior of graphene and aligns qualitatively with previous MD simulations that reported Δ*T* = −27 K for pristine graphene^30^ and Δ*T* = +3.94 K for a similar GK design.^36^ The quantitative difference can be attributed to methodological factors such as the choice of interatomic potential and the maximum applied strain. Moreover, the deformation snapshots in Figure 2c–f reveal that while pristine graphene remains relatively planar under tensile loading, the GK structure develops pronounced out-of-plane deflections, also consistent with previous observations.^46^ Additional deformation snapshots of the highest-ECC performers are provided in Figure S2.

**Figure 2:**
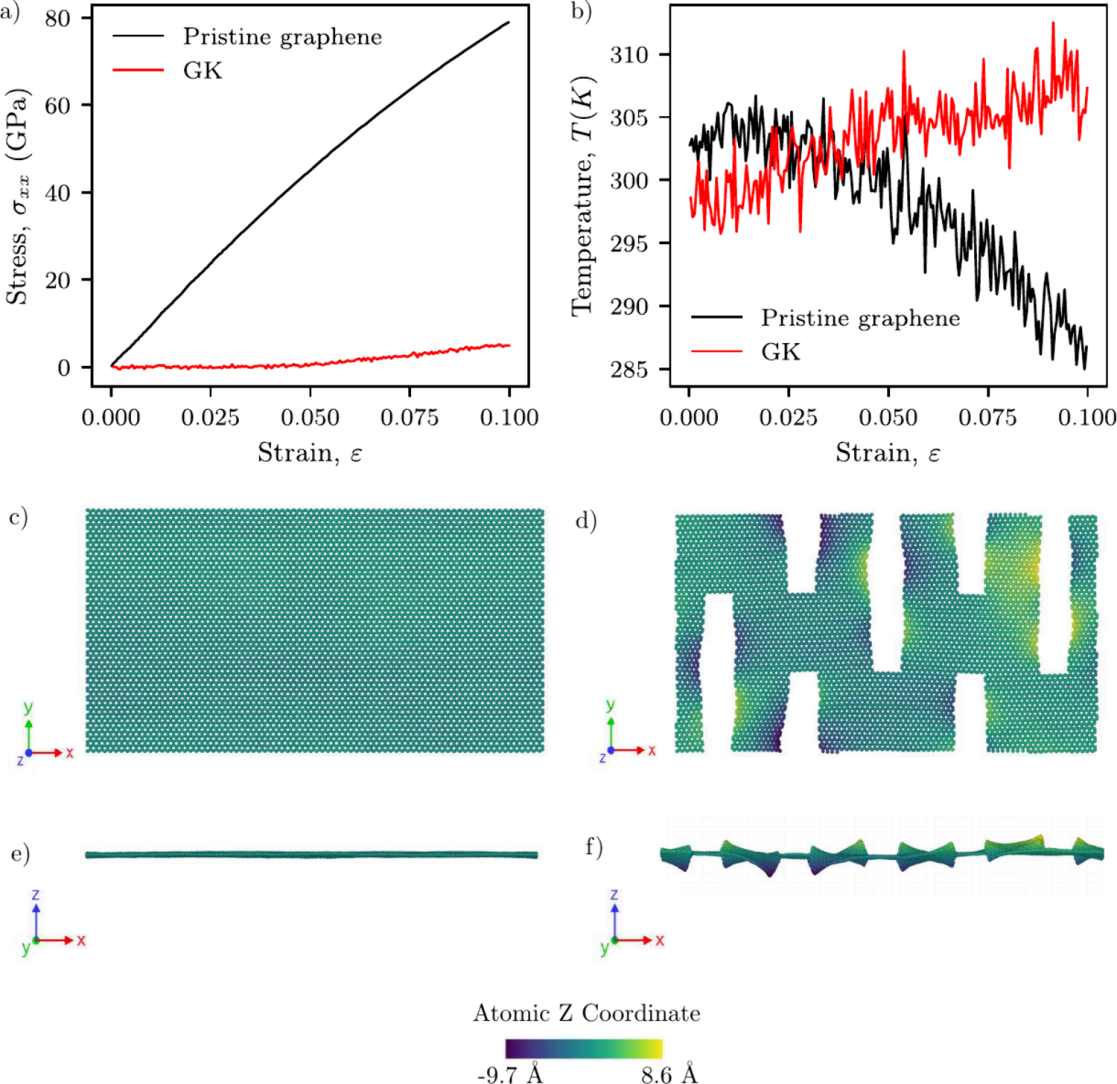
Comparison between pristine graphene and a selected GK. Panels a and b show the stress–strain and temperature–strain curves. Panels c–f show top and side views at 10% strain colored by out-of-plane displacement (center of mass of the structure at *z* = 0).

From the simulation data, we identified correlations among the structural, mechanical, and thermal responses. Figure 3a shows the relationship between temperature variation and toughness (defined as area under the stress–strain curve^47^), with point color indicating defect density. A negative correlation (*R* ^2^ = 0.63) emerges, revealing that structures with lower toughness—typically associated with higher defect concentrations—tend to exhibit higher positive temperature changes, indicative of an enhanced ECE. The lower toughness observed is compatible with previous studies on graphene and hexagonal boron nitride kirigami.^46,48^ Notably, only 42 structures (3.7% of all canonical designs), including pristine graphene, displayed negative Δ*T* values.

**Figure 3:**
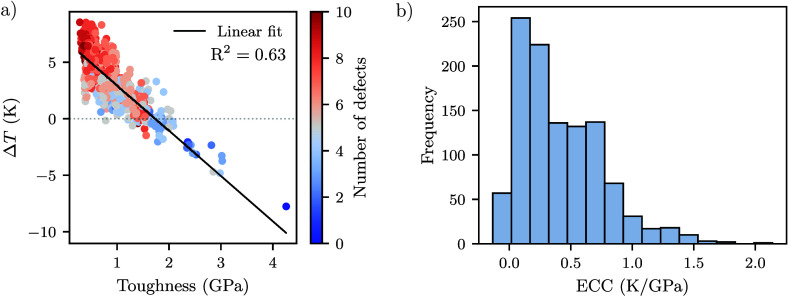
(a) Scatter plot of the temperature variation (Δ*T*) versus toughness, colored by defect density. (b) Distribution of the ECC across all designs.

The distribution of the obtained ECCs, shown in Figure 3b, offers further evidence of the relationship between thermal and mechanical behavior. The ECC values follow a strongly right-skewed distribution, with a mean of 0.42 K GPa^–1^ and standard deviation of 0.34 K GPa^–1^. ECC values spans from −0.14 K GPa^–1^ (pristine graphene) to 2.14 K GPa^–1^, with most designs showing modest ECC values (<0.3 K GPa^–1^), but a small subset exceeds 1.5 K GPa^–1^, emphasizing that specific kirigami geometries can drastically amplify the elastocaloric response.

In order to predict the ECC of GK configurations, we framed the problem as a supervised regression task and employed traditional and deep-learning models using the 3 × 5 array representation of the GK as inputs, with 1 denoting a defect and 0 a pristine cell. The data set was divided into three subsets: 70% for training, 10% for validation (model evaluation and hyperparameter tuning), and 20% for the final testing. Linear regression, random forest, gradient boosting (XGBoost), and multilayer perceptron (MLP) utilized a flattened vector of the GK matrix representation, while for convolutional neural networks (CNNs) the 2D array representation was preserved to exploit local spatial patterns. Hyperparameter optimization was conducted using the optuna package^49^ with 5-fold cross-validation. Traditional ML models were implemented using scikit-learn,^50^ while for the XGBoost regressor,^51^ we employed implementation by DLMC (Distributed Machine Learning Community). Deep-learning models were built in Keras with a TensorFlow backend.^52^ To assess the performance of the ML models, we evaluated the root-mean-squared error (RMSE) and coefficient of determination (*R* ^2^) on the test data set as shown in Table 1.

**Table 1:** Metrics of Performance: RMSE and *R* ^2^ of ML and Deep-Learning Models on the Test Dataset

Model	RMSE (K GPa^–1^)	*R* ^2^
Linear regression	0.249	0.46
Random forest	0.179	0.72
XGBoost	0.138	0.83
MLP	0.127	0.86
CNN	0.064	0.96

Among the tested regressors, the CNN achieved superior accuracy (*R* ^2^ = 0.96), demonstrating the importance of spatially features in determining the ECC response. Hyperparameter optimization was carried out to identify the best-performing CNN architecture. The search space included the number of convolutional filters (from 8 up to 32 filters), the number of convolutional layers (from 1 up to 4 layers), the size of the fully connected layer (from 8 up to 64 neurons), and the dropout rate (from 0 up to 40%). CNN architecture consisted of two convolutional layers with 8 and 12 filters, both using ReLU activation, 3 × 3 kernel size, and periodic padding. The convolutional output was flattened and passed through a fully connected layer with 18 ReLU neurons, followed by a dropout layer (30%) and a single linear output neuron. Model training was performed using the Adam^53^ optimization algorithm, while the mean squared error was adopted as the loss function. The quality of the predictions in different ECC intervals was quantified by the error analysis across quintiles of the true ECC distribution, presented in Table 2.

**Table 2:** Error Metrics of the Best-Performing CNN Model across Quintiles of the ECC^a^

quintile	ECC interval	RMSE (K GPa^–1^)	MAE (K GPa^–1^)	MAPE
1	[−0.135, 0.119]	0.055	0.041	2.221
2	[0.119, 0.254]	0.067	0.054	0.300
3	[0.254, 0.447]	0.072	0.054	0.161
4	[0.447, 0.694]	0.060	0.048	0.083
5	[0.694, 2.140]	0.092	0.074	0.080

a The metrics include root-mean-squared error (RMSE), mean absolute error (MAE), and mean absolute percentage error (MAPE).

The RMSE and MAE remain consistently low across the first four ECC quintiles, indicating uniform predictive accuracy throughout most of the data range. A moderate increase in both metrics appears in the highest quintile, reflecting the lower representation of high-performance structures in the data set. The MAPE complements this trend: its larger values in the lowest quintiles arise from the small magnitude of the true ECCs, while it decreases and stabilizes in higher bins, revealing smaller relative errors for strong elastocaloric responses despite their larger absolute deviations. Figure 4 compares the predicted and true ECC values on the test set, revealing a strong overall consistency, with data points closely following the diagonal, consistent with the error trends summarized in Table 2.

**Figure 4:**
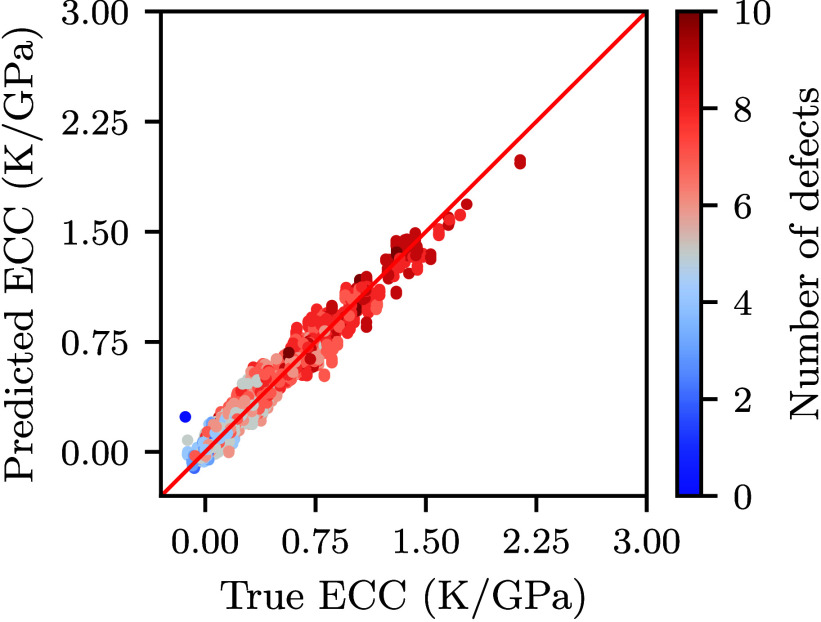
Predicted versus true field-normalized ECC for the CNN model on the test set.

As an application of this best-performing CNN architecture, we verified whether this model could be employed to efficiently search the available design space for structures with high-ECC. As a baseline, we consider a purely random search strategy. In each generation of the search, 300 structures are randomly selected from the available pool and added to the data set. We then compute the mean ECC of the top 50 structures found so far. The random search required 54 generations, corresponding to almost total exploration of the entire data set, to converge to the true mean ECC of the top 50 structures, which is 1.86 K GPa^–1^.

In contrast, our accelerated search begins with the same 300 structures used in the random search. A CNN corresponding to the best-performing architecture from the previous section, is trained on this initial set. The model predicts the ECC for all remaining structures, then 300 structures with the highest predicted values are selected in each subsequent generation, and the process is repeated. The accelerated search significantly outperforms the random baseline as shown in Figure 5. It reaches the true mean ECC of the top 50 structures in only 5 generations, which is approximately ten times faster than the random search. These results indicate that ML-guided search can be an effective tool for identifying optimal kirigami designs in large and complex design spaces. Our results are in agreement with the application of similar strategies for optimizing other mechanical and thermal properties of nanomaterials.^37,54^

**Figure 5:**
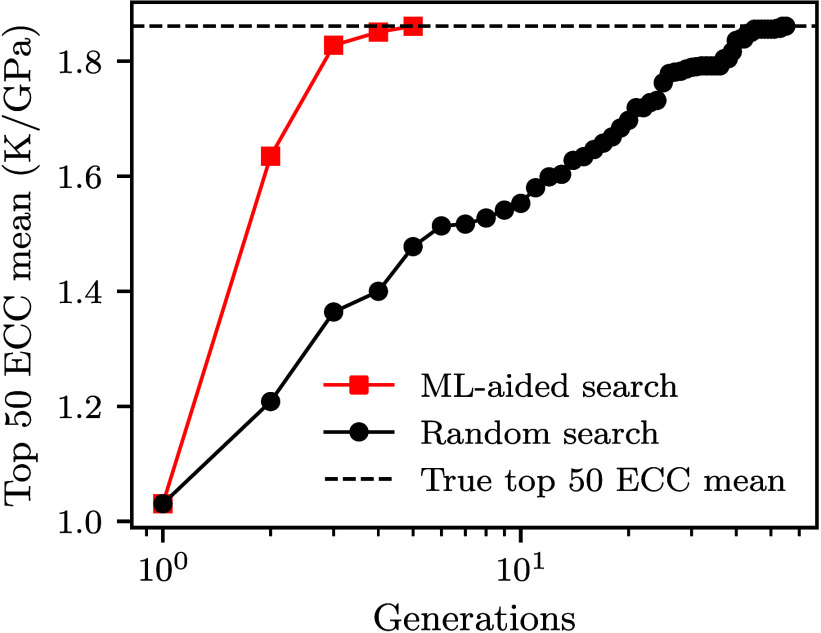
Comparison between the accelerated CNN-guided search and the random search for identifying high-ECC GK structures. The *y* axis represents the mean ECC of the top 50 structures found so far, and the *x* axis corresponds to the search generation.

In summary, we demonstrated an integrated MD–ML framework to accelerate the discovery of GK architectures with enhanced elastocaloric performance. By systematically exploring a comprehensive data set of GK configurations, we revealed clear structure–property relationships linking geometric design to thermomechanical response. Our CNN accurately predicted the ECC from design features from the GK alone, and its use in a guided search efficiently identified high-performing structures an order of magnitude faster than random exploration. These results highlight the potential of data-driven approaches to uncover optimal nanoscale architectures for next-generation solid-state cooling and energy conversion applications.

## Supplementary Material



## Notes

Additional simulation results used to probe the influence of system size and boundary conditions on the computed ECC, and images of the five GK structures exhibiting the highest ECC. The Supporting Information is available free of charge at https://pubs.acs.org/doi/10.1021/acs.nanolett.5c05140.
