# Supplementary material for: Accelerated Discovery of Graphene Kirigami with an Enhanced Elastocaloric Effect via Machine Learning
Source: Nano Lett. 2026 Jan 19;26(4):1267–73. doi: 10.1021/acs.nanolett.5c05140 (PMC12879932; doi:10.1021/acs.nanolett.5c05140)
Supplement: Supplementary file 1 [file nl5c05140_si_001.pdf]

Supporting Information for: Accelerated Discovery of Graphene Kirigami with Enhanced Elastocaloric Effect via Machine Learning

Franklin F. da Silva Filho* and Luiz Felipe C. Pereira*

Departamento de Física, Centro de Ciências Exatas e da Natureza, Universidade Federal de Pernambuco, Recife 50670-901, Brazil

E-mail: franklin.ferreira@ufpe.br; luiz.cpereira@ufpe.br

S1. System Size and Boundary Conditions

To examine the influence of system size and boundary conditions on the computed elastocaloric coefficient (ECC), we evaluated both pristine graphene and a representative graphene kirigami (GK) geometry (Figure S1) using two simulation domains: the size adopted throughout this study (117×200 Å) and a system twice as large (234×400 Å). For periodic boundary conditions, the simulations were performed following the same protocol described in the main text, and the resulting ECC values are reported in Table S1.

Finite-boundary simulations were carried out by constraining all atoms along the left edge of the structure and imposing a constant velocity on the atoms at the right edge, corresponding to the same global tensile strain rate employed in the periodic simulations. The ECC was extracted using the same thermodynamic procedure as in the main calculations, enabling a direct comparison between finite and periodic conditions, as presented in Table S1.

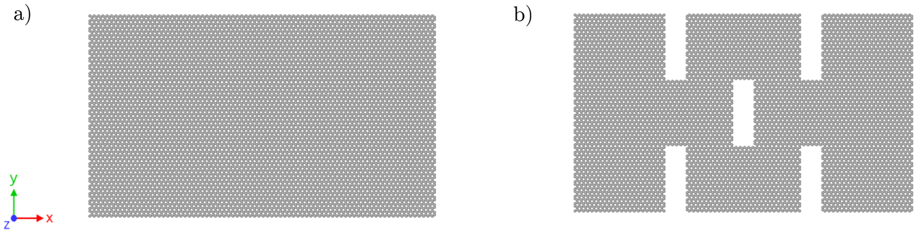

Figure S1: Pristine graphene (a) and a representative graphene kirigami (b) geometry used to assess the influence of system size and boundary conditions on the computed elastocaloric coefficient (ECC).

Table S1: Comparison of Elastocaloric Coefficient (ECC) in Pristine and Kirigami Graphene under Different Boundary Conditions and Sheet Sizes.

**Boundary Conditions**	**Material**	**Sheet Size (Å)**	**ECC (K/GPa)**
Periodic	Pristine	117×200	-0.15 ± 0.02
		234×400	-0.13 ± 0.04
	Kirigami	117×200	0.26 ± 0.09
		234×400	0.31 ± 0.07
Finite	Pristine	117×200	-0.26 ± 0.03
		234×400	-0.26 ± 0.01
	Kirigami	117×200	0.21 ± 0.27
		234×400	0.36 ± 0.16

S2. High ECC structures

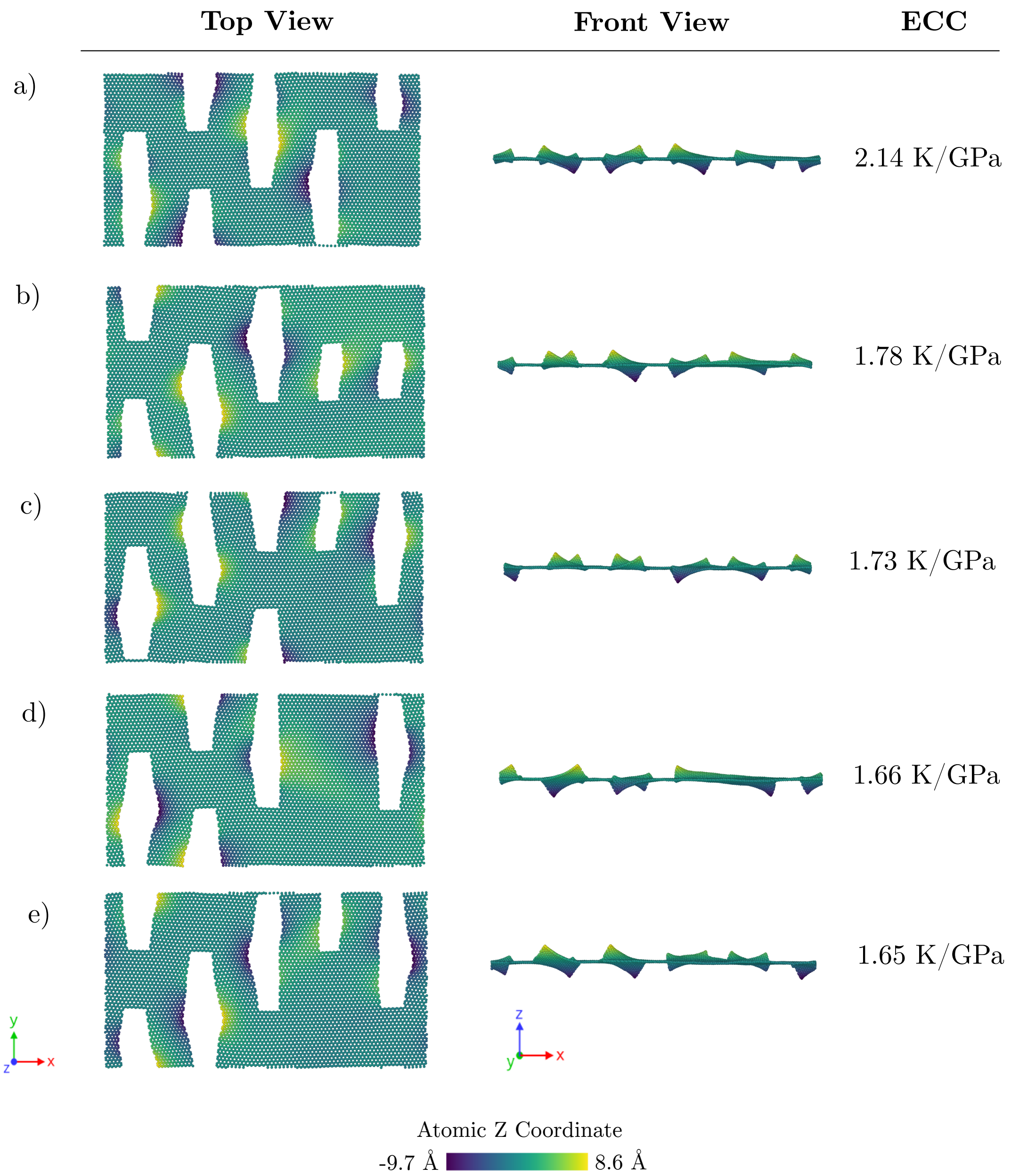

Figure S2: Top and front views of the five graphene kirigami structures exhibiting the highest elastocaloric coefficient (ECC), shown together with their corresponding ECC values.
